# The use of clamped drainage to reduce blood loss in total hip arthroplasty

**DOI:** 10.1186/s13018-015-0259-8

**Published:** 2015-08-25

**Authors:** Jian-gang Cao, Lei Wang, Jun Liu

**Affiliations:** Department of Sport Injuries and Arthroscopy, Tianjin Hospital, Tianjin, 300211 China; Center of Joint Diseases, Tianjin Hospital, Tianjin, 300211 China

**Keywords:** Total hip arthroplasty, Clamped drainage, Blood loss, Wound complications

## Abstract

**Background:**

Drainage is a routine practice used to reduce hematoma and blood loss following total hip arthroplasty. The aim of this study was to assess the effect of clamped drainage on blood loss and wound healing after total hip arthroplasty.

**Methods:**

A prospective cohort of 44 patients with hip osteoarthritis or femur head necrosis undergoing total hip arthroplasty was randomized equally into two groups: 6-h postoperative clamped or non-clamped suction tube drainage. Body mass index, gender distribution, preoperative hemoglobin, hip pathology, and affected side were comparable between the two groups. Blood loss, hemoglobin levels, and wound healing complications were recorded and compared between groups.

**Results:**

The drainage blood loss and calculated blood loss volumes were higher for the non-clamped group. About 100 mL more blood loss was noticed in the non-clamped group. There was no significant difference in adverse events or need for transfusion.

**Conclusions:**

The present study showed a statistically significant reduction in postoperative drainage amount between clamped and unclamped drainage groups, but this difference was not large enough to warrant increased blood transfusion requirements in patients with unclamped drainage. Further studies are essential to define the critical period of clamping that is compatible with the dual objectives of reduced blood loss and lack of wound complications from hematoma.

## Introduction

Total hip arthroplasty (THA) is a successful and widely used method to treat hip disorders such as hip osteoarthritis (OA) and femoral head necrosis.THA can relieve pain, correct deformity, and restore and improve joint movement, making it widely performed by surgeons and accepted by patients. Due to the extensive soft tissue and bone dissection, patients undergoing THA are prone to between 1000 and 2000 mL of blood loss [1–3]. Hematomas are inevitable to a certain extent as complete hemostasis is difficult when the medullary canal is exposed, increasing wound tension, decreasing tissue perfusion, resulting in anemia, and providing an ideal medium for bacterial culture, all of which work against wound healing. Prolonged drainage may lead to a longer hospital stay, increasing the risk of deep vein thrombosis (DVT) and subsequent surgical procedures, and increasing the healthcare economic burden [4]. Waugh and Stinchfield reported lower infection rates when drainage was used, and closed-suction drainage systems, which theoretically decrease the risk of hematoma formation and wound tension, are used as a routine procedure to decrease the possibility of prolonged wound drainage or infection [5]. Koyano et al. evaluated peri-wound skin temperature by thermography, finding that skin temperature was lower in the drainage side, which suggested that drainage can suppress inflammation [6]. In turn, this may promote wound healing and functional joint recovery [7]. Conversely, not all the studies have found similarly promising improvement, with differences not observed between groups with or without closed-suction drainage [5, 8–10]. With results on the use of drainage remaining controversial, accurate information is required in order to fulfill patient expectations and improve outcomes [11–12]. It has been reported that clamping drainage can reduce blood loss and the need for blood transfusion after total knee arthroplasty (TKA) [13]. No similar reports concerning the application of clamping drainage in THA could be found. Therefore, this study was conducted to clarify whether clamping drainage for 6 h postoperatively can contribute positively to the postoperative management of cementless THA, by reducing blood loss and minimizing postoperative wound complications.

## Materials and methods

A randomized controlled prospective study was conducted. Our research had ethical approval by Tianjin Hospital Ethical Approval Committee, with written informed consent about the benefits and risks obtained from all patients. Patients with hip OA or femur head necrosis who were subjected to unilateral primary total hip replacement between July 2012 and January 2014 were included. Exclusion criteria included previous history of hip surgery, ongoing infection, and medical conditions contraindicating surgery. Using a random number list, which was generated by computer and offered to every patient, we randomly allocated patients who meet inclusion criteria to either the flexion group or the extension group. The surgeon who provided surgery did know whether the drainage tube was clamped or not. Tube clamping was finished by another member of the study. Patients did not know why their drainage tube was clamped or not. All agents for anticoagulation and antiplatelet were changed to low molecular weight heparin 5 days before admission. The anticoagulation was terminated 1 day before surgery. All patients received THA at the lateral decubitus position via posterolateral approach under spino-epidural anesthesia performed by the same group of surgeons. Cefazolin (1.0 g) was used as routine antibiotic prophylaxis 30 min prior to skin cut. Cementless hip prostheses (Link, Hamburg, Germany) were used. Short external rotator muscles were closed after the surgical procedure and the wound closed with a suction tube placed under the deep fascia. At this point, the patients were randomized into either the non-clamped drainage group or the clamped drainage group based on formal randomization and concealed allocation by another researcher, and the suction tube of the patients in the clamped drainage group was clamped for the first 6 h postoperatively. Venous thromboembolism prophylaxis was carried out in the form of subcutaneous low molecular weight heparin (0.4 mL) during the remaining hospital stay, and the first dose was administered 12 h postoperatively. Further mechanical prophylaxis consisted of foot pumps and below-knee thromboembolic deterrent stockings. NSAID-based analgesia was used to control postoperative pain. The surgical wounds and drain sites were assessed daily for the presence of persistent bleeding and serous drainage. Indomethacin 25 mg three times daily was administered to all patients to prevent heterotopic ossification. The drainage tube was removed 24 h postoperatively in both groups. Ankle pumping and quadriceps-setting exercises were encouraged immediately after surgery and extension–flexion movement was performed under the supervision of a physical therapist after removal of the tube. Data collection was then conducted by medical students who were blinded for the study purposes. Blood loss parameters were the main results of interest. The hemoglobin (Hb) level was measured preoperatively and 48 h postoperatively. Based on the maximum perioperative decrease in Hb level, total blood loss was calculated according to Gross [14]. Drainage blood was recorded when the tube was removed 24 h postoperatively. Homologous blood transfusion was performed according to a trigger of Hb level less than 9 g/dL. Adverse events during wound healing and recovery were recorded, including hematoma, redness of the incision of more than 1 cm, superficial or deep infection, and DVT during hospital stay or within the initial 6-week postoperative period. Deep infection was diagnosed based on a positive culture from the wound. Any clinical suspicion of DVT was investigated and confirmed by a Doppler ultrasound. Statistical analysis was conducted with SPSS v. 17.0 (SPSS Inc., Chicago, IL, USA) statistical software. Continuous data was expressed as mean ± standard deviation and tested with Student’s tests for difference. Categorical data was analyzed using chi-square tests. A two-sided *p* < 0.05 was considered to be statistically significant.

## Results

A total of 44 patients were recruited into the study. There were 15 women and seven men in the clamped group, with mean age of 73.6 ± 5.5 years and with a body mass index (BMI) of 26.8 ± 2.3 kg/m^2^. The non-clamped group consisted of 14 women and eight men with a mean age of 72.6 ± 4.6 years and BMI of 27.3 ± 2.7 kg/m^2^. Hip pathology was similar in both groups, consisting of 14 cases with OA and eight with femur head necrosis in the clamped group, and 16 OA and six femur head necrosis in the other group. Affected side was left for 10 cases and right for 12 in the clamped group, and left for nine cases and right for 13 in the non-clamped group. Preoperative Hb was 14.5 ± 2.0 and 14.1 ± 2.4 g/dL for the clamped and non-clamped groups, respectively. There was no statistically significant difference between the two groups for these parameters. Drainage blood was recorded, and the Hb level was tested 24 h after tube removal. Drainage blood measured was 177.3 ± 18.4 and 252.2 ± 50.9 mL in the clamped and non-clamped groups (*p* = 0.000), with Hb 11.8 ± 1.6 and 10.8 ± 1.3 g/dL (*p* = 0.033) for each group. The calculated blood loss volumes were 1090.3 ± 124.2 mL for the clamped group and 1223.0 ± 134.0 mL for the non-clamped group (*p* = 0.002). Transfusion was performed for one patient in the clamped group and three patients in the non-clamped group which was not a significant difference (*p* = 0.293). For adverse events, there was no serious deep infection or DVT in any cases in either group. One superficial infection, two hematomas and three redness of incision were observed in the non-clamped group, and two superficial infections, four hematomas, and two redness of incision in the clamped group. No statistically significant difference between the two groups was found for adverse events (*p* = 0.571).

## Discussion

As in TKA, the effect of closed-suction drainage on blood loss and wound complication has been debated in total hip arthroplasty. Some randomized studies and meta-analysis have not supported the routine use of drainage [10, 12, 15]. Kim et al. did not find a specific correlation between the incidence of wound complication and the use or non-use of closed-suction drainage [7]. Different types of drainage have been analyzed in TKA with promising effects on reduction of blood loss and the need for blood transfusions [13, 16]. Release of the tourniquet has significant influence on the blood flow in a short time after the reestablishment of blood flow [17]. Therefore, clamping for a short period can provide the same effect as persistent drainage under the compression of an elastic bandage after tourniquet release [16]. THA differs from TKA as it is not possible to compress the wound and use a tourniquet. Local pressure acts to stop blood loss, and clamping drainage postoperatively may result in greater pressure, contributing to hemostasis [18]. Similarly to TKA, the ideal duration of clamping is unclear from the literature, although a longer clamping period is associated with increased problems such as delayed wound healing, skin edge necrosis, hematoma, and increasing risk of infection [16, 19]. An increase in wound problems or postoperative complication was not observed in the clamped group compared with the non-clamped group. An important focus is the balance of reducing blood loss and preventing local accumulation. The most ideal result is less blood loss and need of blood transfusion without increasing complications of the wound healing resulting from hematoma. In our study, 6-h clamping period can reduce postoperative blood loss by 100 mL without more wound problems. However, the need of blood transfusion was not statistically different. More clamping time may be tested such as 8 or 12 h for the effect of tube clamping in future studies. Limitations to this study include the short postoperative period under study. As no power calculation was undertaken at the start of this study, there is a high risk of the results being underpowered However, the results were trustable because this study was designed well with high methodological quality and they were discussed honestly. Hip functional recovery was not evaluated, despite reports of higher postoperative Hb levels correlating with better early functional recovery, higher SF-36 scores, higher patient satisfaction, and shorter hospital stays following THA [6, 14]. No consensus about the effect of different THA types, such as cemented or hybrid, on total blood loss exists, and the effect of clamping [20–22], and limited by the small patient numbers, this study was unable to compared different types of THA. Further studies regarding the benefit of postoperative clamping drainage in patients following differing types of THA, and length of time of clamping, would be useful.

## Conclusions

This study was performed in order to establish if postoperative clamping had an effect on blood loss, postoperative complications, and Hb levels after cementless THA. The results suggest that clamped drainage for 6 h postoperatively can reduce postoperative blood loss, without an increase in wound healing complications or other postoperative complications, compared with non-clamped drainage. Although it presented a significant difference, the decrease of blood loss was relatively small and no significant difference in need of blood transfusion, suggesting that the effect of postoperative clamping should be questioned for this type of THA. Further investigation into length of clamping time and the effect of clamping with differing THA types could further inform practice, as well as the effect of clamping on long-term functional outcomes.
